# Endocrine Disruption of Propylparaben in the Male Mosquitofish (*Gambusia affinis*): Tissue Injuries and Abnormal Gene Expressions of Hypothalamic-Pituitary-Gonadal-Liver Axis

**DOI:** 10.3390/ijerph20043557

**Published:** 2023-02-17

**Authors:** Yun Ma, Yujing Li, Xiaohong Song, Tao Yang, Haiqin Wang, Yanpeng Liang, Liangliang Huang, Honghu Zeng

**Affiliations:** 1 College of Environmental Science and Engineering, Guilin University of Technology, Guilin 541000, China; 2 Guangxi Key Laboratory of Environmental Pollution Control Theory and Technology, Guilin University of Technology, Guilin 541000, China; 3 Collaborative Innovation Center for Water Pollution Control and Water Safety Guarantee in Karst Area, Guilin 541000, China

**Keywords:** propylparaben, male mosquitofish, HPGL axis, tissue injuries, gene transcriptions

## Abstract

Propylparaben (PrP) is a widely used preservative that is constantly detected in aquatic environments and poses a potential threat to aquatic ecosystems. In the present work, adult male mosquitofish were acutely (4d) and chronically (32d) exposed to environmentally and humanly realistic concentrations of PrP (0, 0.15, 6.00 and 240 μg/L), aimed to investigate the toxic effects, endocrine disruption and possible mechanisms of PrP. Histological analysis showed time- and dose-dependent manners in the morphological injuries of brain, liver and testes. Histopathological alterations in the liver were found in 4d and severe damage was identified in 32d, including hepatic sinus dilatation, cytoplasmic vacuolation, cytolysis and nuclear aggregation. Tissue impairments in the brain and testes were detected in 32d; cell cavitation, cytomorphosis and blurred cell boundaries appeared in the brain, while the testes lesions contained spermatogenic cell lesion, decreased mature seminal vesicle, sperm cells gathering, seminiferous tubules disorder and dilated intercellular space. Furthermore, delayed spermatogenesis had occurred. The transcriptional changes of 19 genes along the hypothalamus–pituitary–gonadal–liver (HPGL) axis were investigated across the three organs. The disrupted expression of genes such as *Ers*, *Ars*, *Vtgs*, *cyp19a*, *star*, *hsd3b*, *hsd17b3* and *shh* indicated the possible abnormal steroidogenesis, estrogenic or antiandrogen effects of PrP. Overall, the present results provided evidences for the toxigenicity and endocrine disruptive effects on the male mosquitofish of chronic PrP exposure, which highlights the need for more investigations of its potential health risks.

## 1. Introduction

Parabens are a group of alkyl esters widely used as ideal preservatives in cosmetics, food and pharmaceuticals since the mid-1920s, due to their excellent antiseptic, antimicrobial and antifungal properties [1]. Parabens are divided into several categories depending on the length of the ester chain, with alkyl substituents ranging from methyl to butyl or benzyl groups [2,3]. These compounds penetrate the environment during production, use and sewage treatment. Parabens are frequently found in tap water, bottled water, rivers, lakes, drinking water and waste water at concentrations from ng/L to μg/L [4,5,6] and have even been detected in aquatic animals [3,7]. According to the assessment of acute and chronic toxic exposure to parabens, they have been classified as emerging contaminants with the capability of endocrine disruption [8,9]. The potential risks of parabens to the environment and to humans have attracted considerable concern and resulted in the “paraben free” campaign [2].

Propylparaben (PrP), with the chemical name Propyl 4-hydroxybenzoate and 4-Hydroxybenzoic acid propyl ester, is one of the most commonly used parabens. The Scientific Committee on Consumer Safety (SCCS) suggested that propylparaben is safe for humans when used as a preservative in cosmetic products (up to a maximum concentration of 0.14%) [10]. While high values of PrP have been reported in human urine samples with a mean concentration at 34.9 μg/L (ND-462 μg/L) [11]. Moreover, PrP was also found in human blood (<0.01–12.1 μg/L) [12], breast milk (ND-0.6 μg/L) [13], maternal urine (0.23–12.44 μg/L) [14,15], urine of newborn infants (0.44–16.9 μg/L) [16], plasma (median: 19.22 μg/L) and amniotic fluid (median: 18.82 μg/L) of pregnant women [17], indicating that the ubiquitous PrP may adversely affect human health and even the developing fetus in the uterus.

PrP is also frequently detected in the aquatic environment, for instance, ND-217 μg/L in the groundwater, ND-229 μg/L in the surface water [8,18,19,20], ND-480 ng/L in source water, ND-590 ng/L in drinking water [21], 23 ng/L in mineral water and 9 ng/L in tap water [6]. It is noteworthy that PrP has the possibility of bioaccumulation in nontarget aquatic organisms, since it had been absorbed by wild fish (0–4.58 μg/kg ww in the muscle, 0–6.72 μg/kg ww in the liver, 0–8.38 μg/L in the plasma) [7,22], so the toxicity of PrP to aquatic organisms can not be ignored.

Previous reports indicated that PrP possesses endocrine interference [23], reproductive toxicity and developmental abnormality [24,25,26] to aquatic organisms. The adverse effects of PrP on the early development of zebrafish was manifested by abnormal changes in hatching rate, heart rate, survival, non-lethal malformations and anxiety-like behavior; these developmental toxicities were associated with increased oxidative stress indices and upregulated expression of apoptotic cells in a dose-dependent manner in the head of zebrafish larvae [27]. It was confirmed that PrP affected the genes from physiological pathways in the 120 dpf zebrafish, including stress response, cell cycle, DNA damage, inflammation, fatty acid metabolism and endocrine functions, and PrP showed an antiandrogenic and estrogenic activity [28]. In the 20 dpf juvenile zebrafish, 45 days of PrP exposure seemed to influence the sex differentiation processes, as the sex ratio significantly skewed towards females [29]. Similarly, the female ratio of the marine copepod (*Tigriopus japonicus*) was increased by 50 μg/L PrP exposure during the development, and males showed higher sensitivity compared to females in the acute toxicity assessment, indicating that PrP had a feminization effect [30].

Both in vitro and in vivo studies suggested that PrP affect estrogenic or antiandrogenic activity, disturb adipogenesis and steroidal sex hormonal homeostasis [31,32]. PrP exposure could activate estrogen-related pathways, for example, 20 nM (3.6 μg/L) PrP stimulated both the mRNA (24 h exposure) and protein (48 h exposure) expression of the progesterone receptor (PGR), estrogen receptor ERα and Erβ in MCF-7 breast cancer cells [33]. In silico molecular docking analyses showed that PrP and other parabens fitted well into the active site of human estrogen receptor ERγ, with hydrogen bonds forming between the *p*-hydroxyl group of parabens and the Glu275/Arg316 of ERγ, and these parabens showed inverse antagonist activities on ERγ, with a lowest observed effect level (LOEL) of 10^−7^ M (18 ng/L) [34]. PrP exhibited significant and concentration-dependent antiandrogenic activity via a yeast-based human androgen receptor assay [35]. Oral doses of PrP had antiandrogenic activity on immature male rats by the supported results of decreased accessory sex organ weights, increasing LH levels and histopathologic changes such as atrophy, hyalinization and anastomosis in androgenic tissues [36]. In addition, PrP disturbed steroid hormones balance by suppressing the serum testosterone level of adult male rats, with a concomitant increase in serum estradiol and an ultimate decrease in testosterone/estradiol ratio [37].

Numerous studies have investigated the endocrine disruption properties of PrP on aquatic organisms, whereas current available information is insufficient. The time- and dose-dependent manner of the potential endocrine effects, and the response of different endocrine related tissues to PrP are not clearly understood; more data are need to elucidate the underlying mechanism. The hypothalamic–pituitary–gonadal–liver (HPGL) axis is a dynamic endocrine system. It maintains the physiological state of reproduction during chemical exposure through various steady-state feedback mechanisms [38]. This axis is associated with gonadotropins release in the hypothalamus and pituitary, yolk protein precursor vitellogenin (VTG) production in the liver, and cholesterol transport and steroidogenesis in gonads of fish [39]. Gene expression changes in the HPGL axis with PrP exposure may reflect disrupted endocrine systems and reproduction of fish; thus, systematic monitoring of genes along the HPGL axis will provide further insights into the reproductive toxicity of PrP.

In this study, the widely distributed mosquitofish (*Gambusia affinis*) was employed as the indicator, and the male fish were exposed to different concentrations of PrP (0, 0.15, 6 and 240 μg/L) for 4 and 32 days, which simulated acute and chronic exposure conditions, respectively. Routine tissue sections (brain, liver and testes) were performed to determine the effects of PrP on the tissues and spermatogenesis in mosquitofish. Related gene expressions in the hypothalamic–pituitary–gonadal–liver (HPGL) axis signaling pathway of corresponding tissues were also investigated to better characterize the acutely and chronically adverse outcomes of PrP on the male mosquitofish.

## 2. Materials and Methods

### 2.1. Fish Care

The mosquitofish were purchased from a local aquaculture farm in Guilin City, China. The fish were domesticated in the aquatic culture system under standard procedures for 14 d, with a pH of 7.1–7.4, a dissolved oxygen ≥5 mg/L, a water temperature of (25 ± 1) °C, and a constant light–dark photoperiod of 14/10 h. They were fed twice a day with commercial fodder and brine shrimp. All animal procedures in this study were conducted based on the guidelines of the Organization for Economic Cooperation and Development (OECD), specifically, the OECD guideline for the testing of chemicals—fish short term reproduction assay (OECD 229), with minor modifications [40]. The experiment was approved by the Animals Ethics Committee of the Guilin University of Technology and the operations were carried out in accordance with the relevant regulations.

### 2.2. PrP Exposure

Propylparaben (PrP, CAS: 94-13-3, purity ≥ 99.0%) was purchased from Xilong Science Co., Ltd. (Guangdong, China). The PrP stock solution was dissolved in Dimethyl Sulphoxide (DMSO), and the concentration of DMSO in the exposure solution and control group was kept with 0.05% (*V*:*V*). According to the 96h-LC_50_ of PrP in mosquitofish (9.14 mg/L based on our previous experiment), environmental levels, and humanly realistic concentrations, four different nominal PrP treatment groups (0, 0.15, 6 and 240 μg/L) (40-fold gradient) were designed with four replicates. These concentrations corresponded to the PrP concentration in the river water (145 ng/L) [6] and surface water (ND-229 μg/L) [8]. The 240 μg/L PrP (about 1/40 96h-LC_50_) represented the highest concentration in the aquatic environment and the median concentration in human fluids (ND-462 μg/L) [11]. Tap water was aerated for more than 48 h and used as diluent of the PrP stock solution in the exposure experiment. The temperature, pH and dissolved oxygen of the exposure solution were monitored daily, and the exposure conditions were maintained to be the same as those of the domestication stage.

A total of 384 healthy male mosquitofish with average lengths of (2.07 ± 0.28) cm and weights of (0.12 ± 0.05) g were selected for the PrP exposure experiment. In each replicated group, 12 male mosquitofish were randomly assigned into a 2 L glass beaker, which was filled with 2 L exposure solution. The exposure period lasted for 32 d and the survival of the tested fish was recorded daily.

Based on the semi-static water change method, 1/2 of the exposure solution was changed every day. The fish were fed twice with commercial fodder and brine shrimp, the residual bait and excrement was fixed and sucked out in time, and the survival of male mosquitofish was recorded daily.

### 2.3. Sample Collection

PrP in the aquatic environment may occur intermittently due to seasonal changes, variable industrial and agricultural activities, thus, aquatic organisms may be affected by periodic peak exposure and chronic exposure. The fish of each concentration and control group were sampled at 4 d (acute exposure) and 32 d (chronic exposure). After being euthanized with MS-222, the brain, liver and testes of male mosquitofish were quickly removed on ice under a microscope. In each glass beaker (4 per concentrations), tissues of three fish were placed in the RNA preservation solution (TaKaRa, Shiga, Japan) and stored at −20 °C for the extraction of total RNA (n = 12 fish in total for each time-point and each PrP concentration). Additionally, tissues of another five fish were immediately fixed in 10% neutral buffered formalin and stored at room temperature for the pathological sections (n = 20 fish in total for each time-point and each PrP concentration).

### 2.4. Tissue Sections and HE Staining

The brain, liver and testes samples of the control and PrP groups were processed for tissue sections and HE staining. Briefly, the samples were dehydrated, fixed in paraffin, sectioned, mounted on glass slides, dried and HE stained. In the HE staining, the samples were fixed with methanol for 10 min, water flushed for 2 min, hematoxylin stained for 2.5 min, water rinsed for 10 min, alcohol hydrochloride differentiated for 2 s, water rinsed for 10 min, eosin stained for 30 s, 70% ethanol washed for 10 s (twice), 80% ethanol washed for 10 s (twice), 90% ethanol washed for 10 s (twice), absolute ethanol rinsed for 10 s and xylene transparented for 10 min. The sections of fish from each group were observed and captured on the 40× objective using the microscope (Nikon ECLIPSE Ti).

The histological analysis of the brain, hepatocytes and testes were performed according to the method described in our previous research [41]. Briefly, the brain structures were identified according to Ullmann [42] and Simões [43]. The identification of hepatocyte lesions was conducted as described by Macêdo [44] and Agamy [45]. The testes structure of mosquitofish were identified according to Leusch [46]. Adult male sperm nests of the mosquitofish contain germ cells at different stages of development, namely, the primary spermatogonium (S1), secondary spermatogonium (S2), primary spermatocyte (S3), second spermatocyte (S4) and spermatozeugmata (Sz). The proportions of germ cells at different developmental stages were analyzed in 100 cells of each fish (n = 20).

The slides of the brain, liver and testes samples were scored semi-quantitatively and classified into four groups based on the average number of each lesion [47,48]. The groups were divided as follows: none or occasional: − (no lesion or 1–2 lesions), mild: + (3–5 lesions), moderate: ++ (6–8 lesions) and severe: +++ (≥9 lesions) [49].

### 2.5. RNA Extraction and qPCR

Total RNA from each sample was isolated using the Trizol reagent kit (TaKaRa, Japan) referring to the manufacturer’s instructions, and RNA quality was determined using a Quawell Q5000 spectrophotometer. The reverse transcription of the total RNA was performed using the PrimeScript reagent Kit with gDNA Eraser (TaKaRa, Japan). A total of 19 target genes related to the hypothalamic–pituitary–gonadal–liver axis were selected to detect the mRNA expression in different samples, including the estrogen receptor genes (*erα* and *erβ*), androgen receptor genes (*arα* and *arβ*) [50], vitellogenin genes (*vtgB* and *vtgC*), cytochrome P450 genes (*cyp19a*, *cyp19a1a*, *cyp19a1b*, *cyp11a1*), steroid 17-alpha-hydroxylase/17,20 lyase (*cyp17*), gonadotropin releasing hormone (*gnrh*), gonadotropin releasing hormone receptor (*gnrhr*), hydroxy-delta-5-steroid dehydrogenase, 3 beta- and steroid delta-isomerase cluster (*hsd3b*), hydroxysteroid 17-beta dehydrogenase 3 (*hsd17b3*), 20β-hydroxysteroid dehydrogenase type (*hsd20b*), sonic hedgehog (*shh*), patched 1 (*ptc1*) as well as the steroidogenic acute regulatory protein (*star*) [51,52]. The glyceraldehyde-3-phosphate dehydrogenase gene (*gapdh*) was served as the endogenous reference gene [53]. The qPCR specific primers are illustrated in Supplementary Material (Table S1).

The real-time PCR experiment was carried out using QuantStudio 3 equipment (Applied Biosystems, Waltham, MA, USA) with PowerUp SYBR Green Master Mix kits (Applied Biosystems). Each qPCR reaction was conducted in triplicate, and each plate included a negative control. According to the operating instructions, the PCR reaction system included 10 μL of PowerUp SYBR Green Master Mix, 2 μL of cDNA template, 1 μL of upstream and downstream primers (10 μmol/L) and 6 μL of ultrapure water. The reaction procedure was set as 95 °C, 30 s; 40 cycles (95 °C, 5 s; 55 °C, 30 s); melting curves: 95 °C, 10 s; 65 °C, 5 s; 95 °C, 0.50 s. The mRNA expression of the target genes were analyzed using the 2^-∆∆Ct^ method [54].

### 2.6. Statistical Analysis and Cluster Heat Map Analysis

The results are presented as the mean ± SEM (standard error of the mean), and data processing and plotting were performed using Graphpad 8.3.0 software. Cluster analysis of all the genes in the three organs were performed to visualize the gene expression patterns across different PrP stressors, using the Origin software with Heat Map with Dendrogram v2.00 tool. The differences in mRNA expressions between the control group and the treatment groups were analyzed by *t*-test and one-way ANOVA (Tukey’s multiple comparison). The statistical significance is considered when *p* < 0.05 and indicated by asterisks (* *p* < 0.05; ** *p* < 0.01).

## 3. Results

No deaths occurred in the male mosquitofish from various treatment groups (0, 0.15, 6 and 240 μg/L) during the 32d PrP exposure, indicating that the chemicals tested at the concentrations in the present study were not acutely toxic.

### 3.1. Brain Histopathology and Gene Expression Changes of HPGL Axis

#### 3.1.1. Brain Histopathology

**Figure 1:**
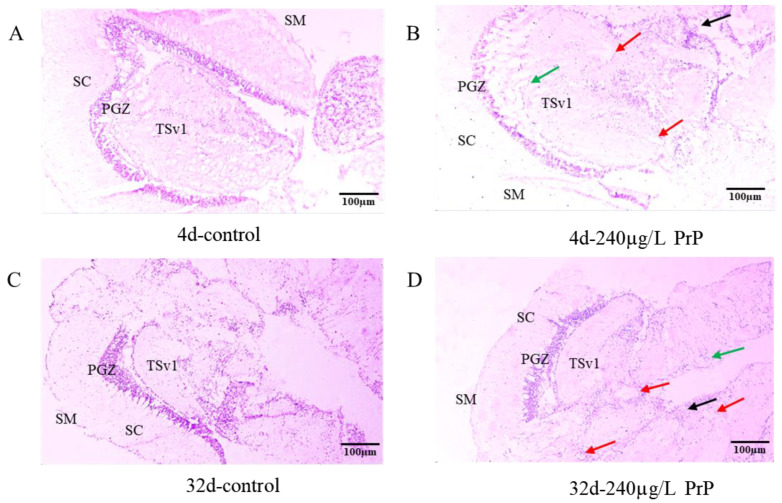

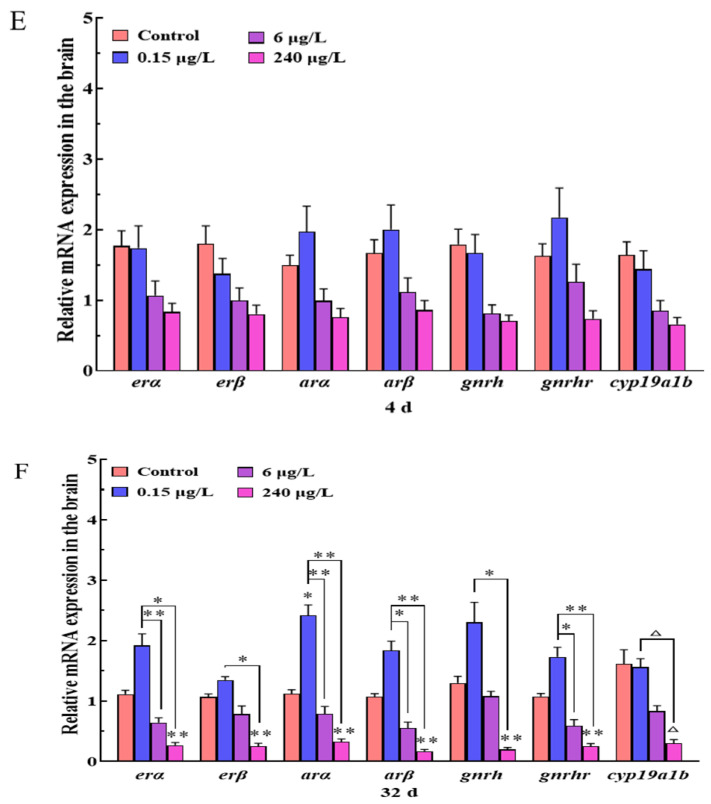
(**A**–**D**): The brain histopathology of male mosquitofish from the control ((**A**): 4d and (**C**): 32d) and PrP-severely affected group (240 μg/L PrP) ((**B**): 4d and (**D**): 32d). Bars = 100 μm, n = 20. Black arrows: blurred cell boundaries; Red arrows: cell vacuolation; Green arrows: cytomorphosis. SM: stratum marginale, SC: stratum centrale, PGZ: stratum periventriculare tecti optici, TSvl: Nucleus ventrolateralis tori semicircularis (ventrolateral nucleus of semicircular torus). (**E**,**F**): Transcriptional levels of HPGL axis related genes in the brain of male mosquitofish suffered from 0, 0.15, 6 and 240 μg/L PrP treatment for 4d (**E**) and 32d (**F**), respectively. Data were analyzed by *t*-test and Tukey’s multiple comparisons. Asterisks (*) above the bars indicate statistical significance (* *p* < 0.05, ** *p* < 0.01, △ *p* < 0.08). *erα*, estrogen receptor alpha; *erβ*, estrogen receptor beta; *arα*, androgen receptor alpha; *arβ*, androgen receptor beta; *gnrh*, gonadotropin releasing hormone; *gnrhr*, gonadotropin releasing hormone receptor; *cyp19a1b*, cytochrome P450, family 19, subfamily A, polypeptide 1b.

In the brain sections, the structures of stratum marginale (SM), stratum centrale (SC) and stratum periventricular tecti optici (PGZ) were shown in Figure 1A–D. The stratum marginale was formed by nerve fibers and a few neurons, the stratum centrale contained more nerve cells, and the periglomerular gray zone (PGZ) contained dense neurons.

After PrP exposure for 4d and 32d, the histological sections showed that the brain tissue in the control group had clear stratification and tight cells (Table 1, Figure 1A,C). The brain damage got worse gradually, following the increase of PrP concentrations and exposure duration. In the 240 μg/L PrP group of 4d and all PrP groups of 32d, severe pathological changes were observed in the brain, including cell cavities, cytomorphosis and blurred cell boundaries (Table 1, Figure 1B,D).

#### 3.1.2. Gene Expression Changes of HPGL Axis in the Brain

After PrP exposure for 4 d, the relative expressions of endocrine-related genes (*erα*, *erβ*, *arα*, *arβ*, *gnrh*, *gnrhr* and *cyp19a1b*) in the brains of all PrP treatment groups (0.15, 6, and 240 μg/L) had no significant difference from that of the control group (*p* > 0.05) (Figure 1E). However, when the PrP exposure was extended to 32 d, the gene transcriptions in the PrP groups displayed in a parabolic path, the transcriptional levels increased in the 0.15 μg/L PrP group and declined in the 240 μg/L PrP group (Figure 1F). The expressions of all the target genes in the 240 μg/L PrP group were significantly lower than that of the control group and the 0.15 μg/L PrP group (*p* < 0.05) (except the *cyp19a1b* gene, *p* < 0.08).

### 3.2. Liver Histopathology and Gene Expression Changes of HPGL Axis

#### 3.2.1. Liver Histopathology

**Figure 2:**
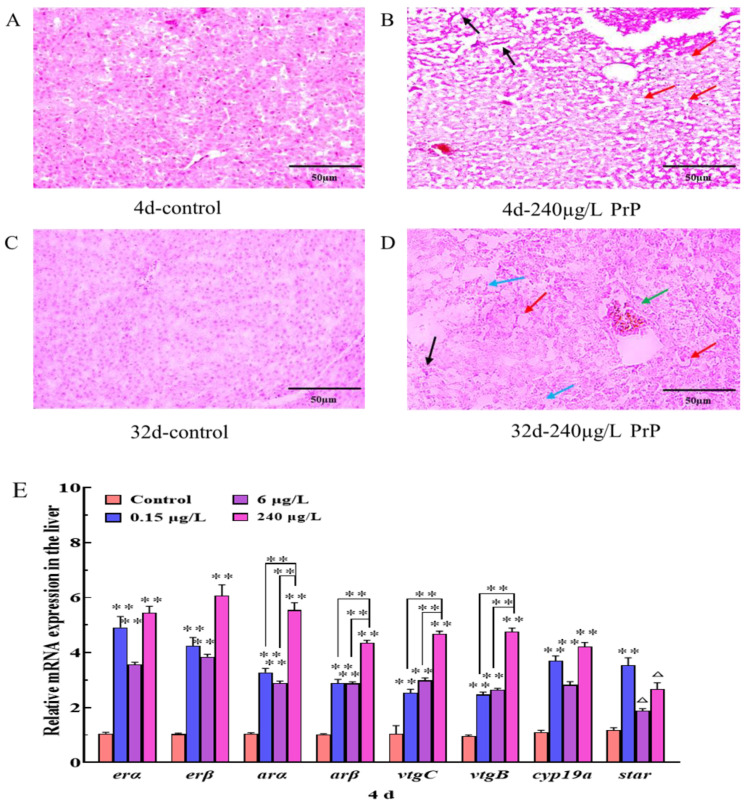

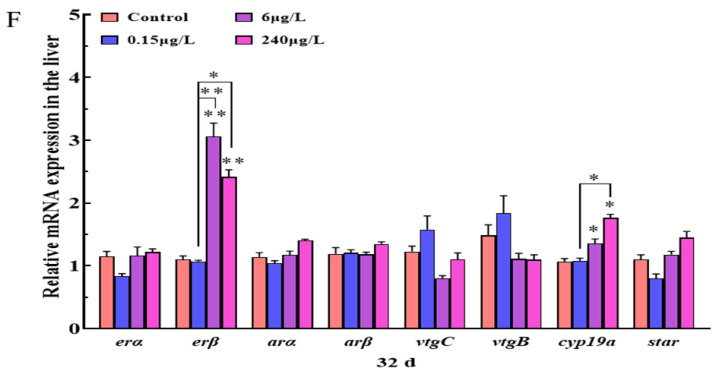
Histological appearance of hepatocytes in the liver of male mosquitofish from the control ((**A**): 4d and (**C**): 32d) and PrP-severely affected group (240 μg/L PrP) ((**B**): 4d and (**D**): 32d). Bars = 50 μm, n = 20. Black arrows: hepatic sinus dilatation; Red arrows: cytoplasmic vacuolation; Blue arrows: cytolysis; Green arrows: nuclear aggregation. (**E**,**F**): transcriptional levels of HPGL axis related genes in the liver of male mosquitofish suffered from 0, 0.15, 6 and 240 μg/L PrP treatment for 4d (**E**) and 32d (**F**), respectively. Data were analyzed by *t*-test and Tukey’s multiple comparisons. Asterisks (*) above the bars indicate statistical significance (* *p* < 0.05, ** *p* < 0.01, △ *p* < 0.08). *erα*, estrogen receptor alpha; *erβ*, estrogen receptor beta; *arα*, androgen receptor alpha; *arβ*, androgen receptor beta; *vtgB*, vitellogenin B; *vtgC*, vitellogenin C; *cyp19a*, cytochrome P450, family 19, subfamily A; *star*, steroidogenic acute regulatory protein.

The histological sections showed that the hepatocytes in the control group were polygonal with clear intercellular boundaries and arranged orderly, round nuclei in uniform sizes were distributed in the center of the cells (Figure 2A,C). PrP induced morphological injuries to hepatocytes in a time- and dose-dependent manner (Table 1, Figure 2B,D), with the prolongation of exposure time and increase concentrations of PrP, the histological injuries in the liver were more severe. Mild and moderate liver injuries were found in the PrP groups (0.15, 6 and 240 μg/L) after PrP exposure for 4 d, mainly including hepatic sinus dilation and cytoplasmic vacuolation. The hepatocellular damage increased in severity after PrP exposure for 32 d, the most common lesions contained hepatic sinus dilation or hyperemia, cytoplasmic vacuoles, nuclear aggregation, cytolysis and partial cell necrosis.

#### 3.2.2. Gene Expression Changes of HPGL Axis in the Liver

In comparison with the control fish, the mRNA expressions of *erα*, *erβ*, *arα*, *arβ*, *vtgB*, *vtgC* and *cyp19a* in the liver were both notably up-regulated after exposure to PrP (0.15, 6 and 240 μg/L) for 4 d (*p* < 0.01 for each case) (Figure 2E). Furthermore, 0.15 μg/L PrP resulted in marked improvement of the *star* gene (*p* < 0.01), and the *star* transcription in the 6 and 240 μg/L PrP groups still kept in a rising trend even though there were no significant alterations (*p* < 0.08). In the 32d PrP treatment cases, all the genes were not affected except the *erβ* and *cyp19a* (Figure 2F). The expressions of *erβ* and *cyp19a* in the 6 μg/L and 240 μg/L PrP groups were significantly higher than that of the control group (*p* < 0.05).

### 3.3. Testes Histopathology and Gene Expression Changes of HPGL Axis

#### 3.3.1. Testes Histopathology and Morphometry

**Figure 3:**
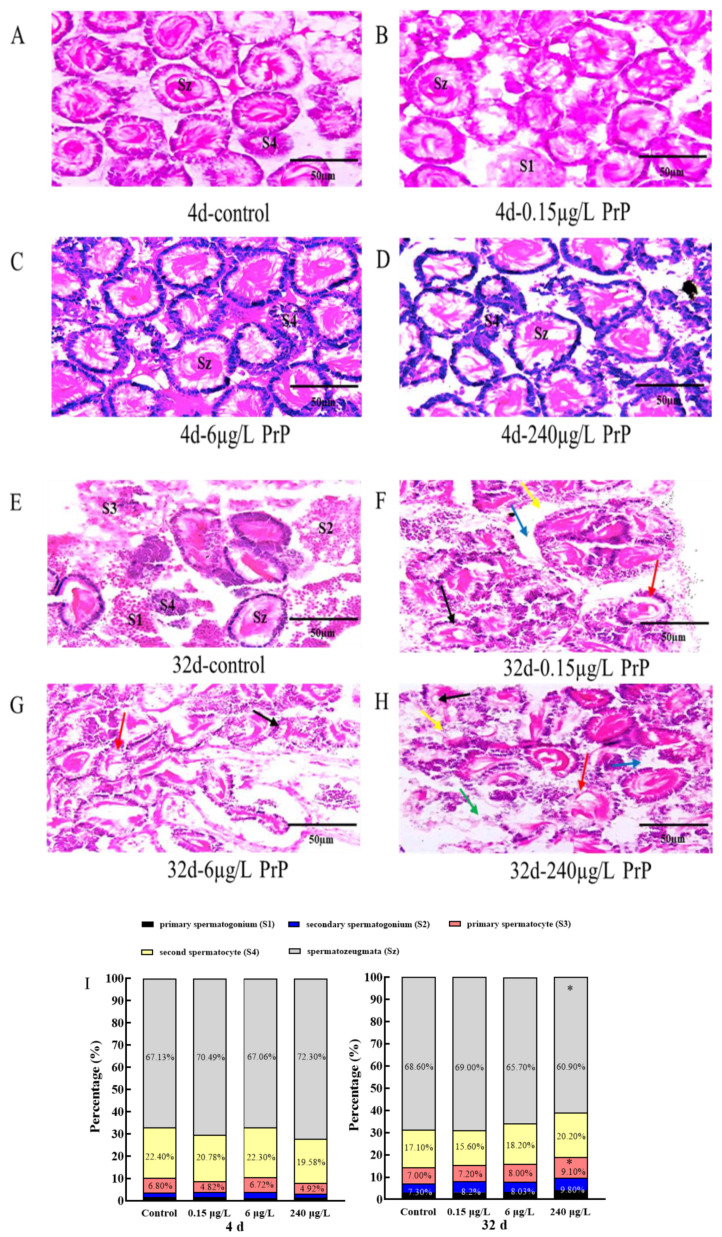
The testes histopathology of male mosquitofish from the control ((**A**): 4d and (**E**): 32d), 0.15 μg/L PrP group ((**B**): 4d and (**F**): 32d), 6 μg/L PrP group ((**C**): 4d and (**G**): 32d) and 240 μg/L PrP group ((**D**): 4d and (**H**): 32d). Bars = 50 μm, n = 20. S1: primary spermatogonium, S2: secondary spermatogonium, S3: primary spermatocyte, S4: second spermatocyte, Sz: spermatozeugmata. Black arrows: spermatorgenic cell lesion; Red arrows: decreased mature seminal vesicle count; Yellow arrows: sperm cells aggregation; Green arrows: seminiferous tubules disorder; Blue arrows: dilated intercellular space. (**I**): Percentage of different germ cells in the male mosquitofish exposed to different concentrations of PrP for 4 d and 32 d. The proportion of germ cells at different developmental stages were analyzed in 100 cells of each fish. Asterisks (*) above the bars indicate statistical significance of the germ cells ratios in the male fish suffered from PrP with the same dose between 4d and 32d.

The testicular development of adult mosquitofish was asynchronous, the testes from all groups contained five kinds of spermatocysts at different developmental stages (S1, S2, S3, S4 and Sz) (Figure 3). In the control group, germ cells at various stages were observed, which were well developed, neatly arranged and in equal sizes (Figure 3A,E). After PrP exposure for 4d, no visible morphological differentiation was found between the control and PrP groups (Figure 3B–D). When the PrP exposure was extended to 32d, the testes seemed to be fragile and disorganized, the tissue damage contained spermatogenic cell lesion, decreased mature seminal vesicle, sperm cells gathering, seminiferous tubules disorder and dilated intercellular space (Table 1, Figure 3F–H).

The proportion of germ cells at different developmental stages were analyzed in 100 cells of each fish. The statistical results showed that when exposed to PrP for 4 d and 32 d, there was no significant difference in the proportion of different sperm cells between the control and PrP treatment groups (*p* > 0.05) (Figure 3I). However, the ratio of mature sperms (Sz) (60.9%) in the 32 d- 240 μg/L PrP group decreased significantly compared to that of 4 d- 240 μg/L PrP group (72.3%), contrarily, the percentage of primary spermatocyte (S3) in the 32 d- 240 μg/L PrP group (9.1%) was higher than that of 4 d (4.92%), which indicated that PrP may induce a delayed spermatogenesis.

#### 3.3.2. Gene Expression Changes of HPGL Axis in the Testes

**Figure 4:**
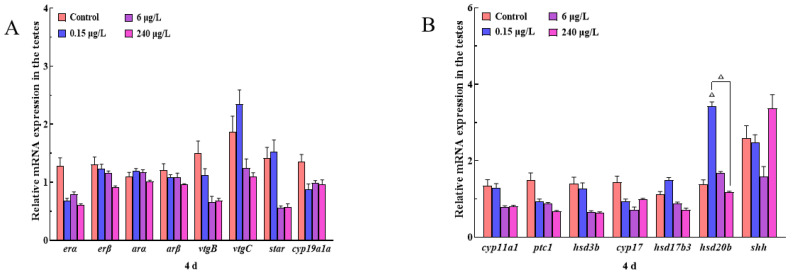

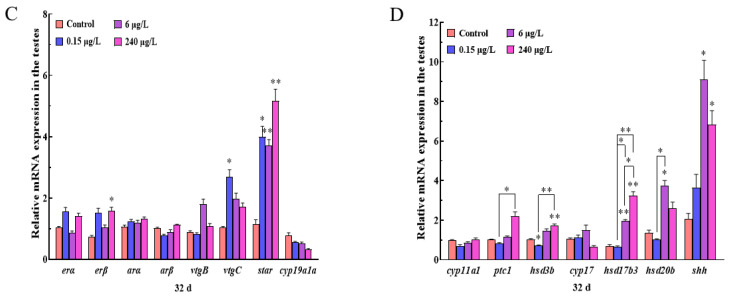
Transcriptional levels of HPGL axis related genes in the testes of male mosquitofish suffered from 0, 0.15, 6 and 240 μg/L PrP treatment for 4d (**A**,**B**) and 32d (**C**,**D**), respectively. Data were analyzed by *t*-test and Tukey’s multiple comparisons. Asterisks (*) above the bars indicate statistical significance (* *p* < 0.05, ** *p* < 0.01, △ *p* < 0.08). *erα*, estrogen receptor alpha; *erβ*, estrogen receptor beta; *arα*, androgen receptor alpha; *arβ*, androgen receptor beta; *vtgB*, vitellogenin B; *vtgC*, vitellogenin C; *star*, steroidogenic acute regulatory protein; *cyp19a1a*, cytochrome P450, family 19, subfamily A, polypeptide 1a; *cyp11a1*, cytochrome P450 family 11 subfamily A member 1; *ptc1*, patched 1; *hsd3b*, hydroxy-delta-5-steroid dehydrogenase, 3 beta- and steroid delta-isomerase cluster; *cyp17*, steroid 17-alpha-hydroxylase/17,20 lyase, *hsd17b3*, hydroxysteroid 17-beta dehydrogenase 3, *hsd20b*, 20β-hydroxysteroid dehydrogenase type, *shh*, sonic hedgehog.

There were no significant changes in 15 endocrine-related genes (*erα*, *erβ*, *arα*, *arβ*, *vtgB*, *vtgC*, *star*, *cyp19ala*, *cyp11a1*, *ptc1*, *hsd3b*, *cyp17*, *hsd17b3*, *hsd20b* and *shh*) in all treatments (0.15, 6 and 240 μg/L) (*p* > 0.05) after PrP exposure for 4d (Figure 4A,B). In the 32d PrP treated fish, the expressions of *vtgC* gene in the 0.15 μg/L PrP group, *hsd20b* gene in the 6 μg/L PrP group, as well as *erβ* gene in the 240 μg/L PrP group were all stimulated (*p* < 0.05). The *star* transcription in all the PrP groups raised sharply (*p* < 0.05). Increased expressions of *hsd17b3* and *shh* were all observed in the 6 and 240 μg/L PrP groups (*p* < 0.05). In the condition of *hsd3b* gene, the gene was down-regulated in the 0.15 μg/L PrP group (*p* < 0.05) while up-regulated in the 240 μg/L PrP group (*p* < 0.01) (Figure 4C,D).

### 3.4. Cluster Heat Map Analysis of HPGL Axis Related Genes in the Male Mosquitofish

**Figure 5:**
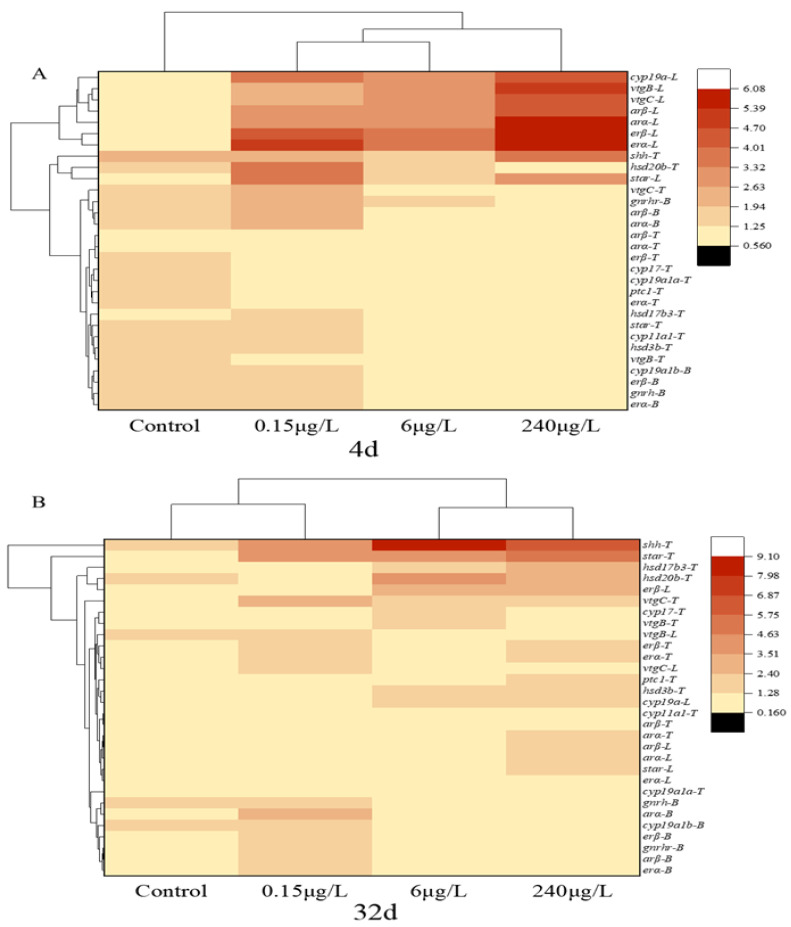
Hierarchical clustering analysis of qPCR data of the male mosquitofish suffered from 0, 0.15, 6, 240 μg/L PrP for 4d (**A**) and 32d (**B**). Abbreviations: B, brain; L, liver; T, testes; other abbreviations for the genes were the same as mentioned above.

In the heat maps, the gene expression data is displayed in a grid where each row represents a gene and each column represents a PrP group. The color and intensity of the boxes represent the gene expression changes. As shown in Figure 5A, the expressions of endocrine-related genes were roughly divided into two categories: upregulation and unaffected. After suffering from different concentrations of PrP for 4d, heatmap analysis revealed that the genes of the three organs (the brain, liver and testes) in the 0.15 μg/L PrP treatment were clustered close to the 6 μg/L PrP treatment, these groups then clustered with the 240 μg/L PrP treatment, all the PrP groups were away from the control group, which indicated that the genes in these treatments shared similar expression patterns. Most of the target genes in the liver with higher expression were clustered on the top, including the *cyp19a*, *vtgB*, *vtgC*, *arβ*, *arα*, *erβ*, *erα* and *star* orderly.

While in the 32d PrP exposure experiments, the genes of the three organs in the 0.15 μg/L PrP treatment were clustered close to the control group, and the genes in the 6 μg/L PrP treatment were clustered together with the 240 μg/L PrP treatment (Figure 5B), which indicated a cumulative effect of exposure time and dose. Most of the target genes in the brain were aggregated at the bottom, the expression of *shh*, *star*, *hsd17b3*, *hsd20b* and *vtgC* in the testes were gathered on the top.

### 3.5. The Time and Dose Dependent Manner of HPGL Axis Related Genes in the Male Mosquitofish

As shown in Figure S1, endocrine-related genes (*erα*, *erβ*, *arα*, *arβ*, *gnrh*, *gnrhr* and *cyp19a1b*) in the brain of the male mosquitofish showed a similar down-regulation expression trend with 4d and 32d PrP treatment at different concentrations. No significant difference was found among all the genes at the two time points with the same concentrations (*p* > 0.05).

Interestingly, the expression trends of *erα*, *erβ*, *arα*, *arβ*, *vtgB*, *vtgC*, *star*, and *cyp19a* in the liver of the 4d-PrP treatments were all up-regulated and higher than that of the 32d-PrP groups (Figure S2). Among these genes, the expressions of *erα*, *erβ*, *arα*, *arβ* and *cyp19a* in all the PrP groups at the two time points were significantly different (*p* < 0.05). In the 6 and 240 μg/L PrP groups, there was a significant difference between the expression of *vtgB* and *vtgC* with different exposure durations (4d and 32d) (*p* < 0.05). Besides, the mRNA level of the *star* gene in the 0.15 μg/L PrP group varied significantly due to different exposure times (*p* < 0.05).

In the testes, the changes of the expression trend were complicated (Figure S3), which indicated that the genes response in the testes were controlled by multiple factors. Statistics analysis showed that some of the tested genes were significantly expressed (*p* < 0.05) with different exposure times, i.e., *hsd20b* at 0.15 μg/L PrP group, *shh* and *star* at 6 μg/L PrP group, *star* and *hsd17b3* at 240 μg/L PrP group. Among these genes, the *star* gene in the 32d-PrP treatments were up-regulated and higher than that of the 4d-PrP groups (6 and 240 μg/L PrP).

## 4. Discussion

### 4.1. PrP Induced Injuries in the Brain, Liver and Testes of Male Mosquitofish

In this study, microstructure observation in the three tissues displayed the overt toxicity of PrP, the mRNA changes of genes along the HPGL axis revealed the cryptic endocrine disruption on the reproductive systems.

The susceptibility to PrP of three target organs in mosquitofish revealed a time-dose response relationship, with the prolongation of exposure time and increase concentrations of PrP, the histological injuries in the brain, liver and testes were more severe. The visible hepatocellular damage occurred in the acute PrP exposure (4d), whereas the lesions in the brain and testes were observed until 32d. Congruously, the transcriptional abnormalities of HPGL axis-related genes in the brain and testes were detected at 32d. While the HPGL axis-related genes tested in the liver were all increased significantly at 4d, only two genes (*erβ* and *cyp19a*) were significantly changed at 32d. Moreover, the expression trends in the liver of the 4d-PrP treatments were all up-regulated and higher than that of the 32d-PrP groups. Thus, the liver was more sensitive to the acute toxicity of PrP, and it seemed to have an adaption of the endocrine dysfunction in the liver. The PrP effects on the brain and testes were not able to be observed immediately, but did occur in the chronic exposure. Therefore, the long-term presence of PrP in the water will threaten the health of aquatic animals, even at low environmental levels.

Parabens can disrupt several molecular pathways within cells via ER-mediated or AR-mediated mechanisms, oxidative stress-induced impairment, lysosomal and mitochondrial disorder and DNA damage [55]. Previous researches demonstrated that PrP induced detrimental influence on zebrafish, including lipid metabolism disorder [26,56], oxidative stress, DNA double-strand breaks and apoptosis [28]. Studies also suggested that PrP and other parabens may contribute to carcinogenicity [55,57]. The negative physiological effects of PrP on the vital tissues of mosquitofish were likely associated with these toxic mechanisms, which may affect normal physiological activities of the brain, liver and testes. The pathological injuries in these tissues were the embodiment of multiple synthetic influences of PrP. The HPGL axis plays a pivotal role in the regulation of reproductive function [58], the gene expression changes along the HPGL axis suggested a compensatory mechanism and feedback-regulation for PrP damage.

As a pivotal organ responsible for detoxification, metabolism, immunization, and epidemic prevention, chemical-induced hazardous effects usually appear primarily in the liver [59]. Available literature revealed that PrP is hepatotoxic. PrP induced disruption of energy metabolism and increased synthesis of superoxide anions and apoptosis in the liver cells [60]. PrP (4.0 mg/L) caused oxidative stress (a decrease in 6d and an increase in 12d of total glutathione) in the liver cells of Nile tilapia (*Oreochromis niloticus*) [61]. Hepatic atrophy and cellular degeneration in the brain were found in the 13 dpf-old medaka eleuthero-embryos exposed to 4000 μg/L PrP during embryo development [25]. In the present work, similar findings were shown in the histological liver structures from PrP groups, which reflected the biochemical changes and failure of cellular protective mechanisms under PrP stress.

It is confirmed that embryonic exposure to PrP triggered anxiety-like neurobehavioral response in zebrafish, which is correlated with oxidative-stress-induced apoptosis in the head of the larvae [27]. Nevertheless, PrP was suggested to reduce the excitability of hippocampal neurons in rats [62], and to have anticonvulsant effects on pentylenetetrazol-induced seizures in zebrafish [63], demonstrating the potential for use in anticonvulsant drugs. In this study, cell cavitation, cytomorphosis and blurred cell boundaries were found in the brain of mosquitofish, the deleterious effects of PrP on the physiological function of brain should be noteworthy to illuminate the safety and toxicity mechanisms.

Numerous studies have verified that PrP exhibits the characteristics of antiandrogenic and estrogenic activity, and has harmful effects on reproductive functions. For the invertebrates, PrP exposure caused fecundity-reduction in the female fruit fly *Drosophila melanogaster* [64], *Aedes aegypti* [65], nematode *Caenorhabditis elegans* [66], and marine copepod *Tigriopus japonicus* [30]. PrP also prolonged the pupation and maturation times of the fruit fly *D. melanogaster* [64]. For the vertebrates, chronic exposure to PrP at humanly relevant doses led to endocrine disorders, altered the estrus cycle, hormone levels and ovarian reserve, as well as accelerated ovarian aging in adult mice [67]. Kolatorova et al. [68] found a negative association between the PrP and testosterone levels in human cord blood, indicating a PrP risk for prenatal male development. PrP exposure resulted in a female-biased sex ratio in juvenile zebrafish [29] and marine copepod *T. japonicus* [30]. Additionally, parabens are suggested as effective spermicides [69], e.g., oral PrP exposure decreased testosterone concentration, sperm production and quality in male rats [70]. However, in Gazin’s research on rats, oral PrP did not show effects on male reproductive organs’ weight, epididymal sperm parameters, hormone levels or histopathology [71].

In the present study, testes lesions and a delayed spermatogenesis were found in the PrP groups, providing evidence that PrP has adverse effects on male reproduction. Similar testes lesions were also found in the zebrafish with methyl paraben exposure, including general testicular atrophy, multi-nucleated gonocytes, impaired germ cell, spermatogonial proliferation, Leydig cell hyperplasia, interstitial fibrosis and apoptosis of Sertoli cells [72]. Teleost gonads are sensitive to environmental factors; endocrine disruptors such as estrogens and estrogen mimics may induce temporary or permanent morphological changes in the gonads and result in an impairment of gonadogenesis and sex differentiation [73]. Steroid hormones and steroid-regulated genes play important and distinct roles in controlling fish spermatogenesis and testis maturation [74]. As expected, there was a robust transcriptional response in the testes of PrP-treated mosquitofish, which help to explain the morphological changes and reproductive abnormality.

### 4.2. Deleterious Impacts of PrP Stress on Endocrine Markers in HPGL Axis

EDCs modulate the endocrine functions through several means; they can bind nuclear receptors as ligands and act like agonists or antagonists, and can disrupt the biosynthesis, metabolism, transportation and biotransformation of endogenous hormones [32]. In this study, most of the target genes associated with the HPGL axis in the male mosquitofish showed significant transcriptional changes during PrP exposure, indicating the endocrine interference potency.

Parabens can act as either an estrogenic agonist or androgenic antagonist. The mRNA expression of several tested genes in the liver of male mosquitofish, including *Ers*, *Ars*, *Vtgs*, *cyp19a* and *star*, were dramatically up-regulated after 4d PrP exposure at all concentrations. These data showed that PrP could disrupt estrogenic and androgenic receptors. Parabens could activate both ERα and ERβ receptors, with similar or stronger effect versus ERβ receptors [2]. Consistently, the *erβ* gene in the liver and testes of the 32d-PrP treated mosquitofish was also significantly increased compared to control. The cytochrome P450 aromatase gene *cyp19a* is a rate-limited step for catalyzing the conversion of androgens into estrogen [75], thereby, the enhanced *cyp19a* transcripts may raise the production speed of estrogen, resulting in the synchronized increase of *erα* and *erβ*. ERs are sensitive biomarker of estrogen endocrine disturbance, similar to other parabens [76,77], the raised *erα* and *erβ* genes at various concentrations and exposure duration confirmed the estrogenic effect of PrP, which may cause imbalance of ERs-dependent transcriptional signaling pathways.

The androgen receptor (AR) is a steroid hormone receptor that is responsible for androgen-sensitive genes regulation, as well as for the development and maintenance of male secondary sexual characteristics [78]. The transcriptional responses of *arα* and *arβ* genes in the liver of PrP exposed group exhibited an upward tendency, indicating an antiandrogenic effect. Similar results were also found in the liver of male medaka that were treated with antiandrogens vinclozolin and flutamide [79]. The steroidogenic acute regulatory protein (*star*) is involved in catalyzing the first step of the steroidogenesis pathway, and play an important role in regulating the transport of cholesterol into the inner mitochondrial membrane [51]. The high *star* transcripts may increase the endogenous androgen levels, which compete with exogenous antiandrogen-like PrP for androgen receptors. Hence, the increased *arα* and *arβ* expressions may be due to the positive feedback results from the increase steroid hormone level of the male fish, displaying a compensatory response for blocking ARs [79].

Vitellogenin (VTG), the fish egg yolk precursor protein, is a common biochemical endpoint to assess the presence of estrogenic substances in fish. The liver is considered to be the main tissue for VTG synthesis in fish [80]. The *vtg* gene is usually silent in male fish but can be stimulated by estrogen and estrogen-like hormones [81]. PrP exposure raised the VTG plasma concentration and mRNA expression levels of *vtg-1*, *vtg-2* and *erα* in the liver of male medaka [82]. Increased plasma vitellogenin levels in the rainbow trout were reported [23,83], which proved that this paraben had oestrogenic properties. Consistent with these studies, significant upregulation of *vtgB* and *vtgC* genes in the liver were found after PrP exposure for 4 d. Meanwhile, the *vtgC* levels in the testes of 32d-PrP groups were higher than that of the control group. ERs are functionally involved in the regulation of vitellogenesis with *Erα* and act as the central mediator in teleost [84]; in the present study, the increased *Ers* genes triggered vitellogenesis and may eventually yield higher contents of VTG. Vitellogenin can cause fertility disorders, such as gonadal histopathology changes or feminization of male fish, which could lead to reproduction suppression of fish [85]. The elevated *vtg* gene expressions may be associated with the proportion changes of spermatogenic cells at various differentiation stages.

Fish reproduction is regulated by synergistic interactions between steroid hormones along the HPGL axis and by steroidogenesis in the gonad [51]. During steroidogenesis, the *star* gene is responsible for the rate-limiting transportation of cholesterol into the mitochondrial membrane, then, the cholesterol is stepwise converted to testosterone under the catalytic action of CYP11a1, 3β-HSD, CYP17a, and 17β-HSD, finally the testosterone is converted to 17β-estradiol by the aromatase (CYP19a) [86]. As known, the hydroxysteroid 3β dehydrogenase (*hsd3b* gene) catalyzes the second step of steroid production, namely converts the pregnenolone to progesterone, which is necessary for the synthesis of all steroids [87]. The hydroxysteroid 17β dehydrogenase 3 (*hsd17b3* gene) is a key enzyme in the last step of sex hormone synthesis, it performs the conversion of androstenedione to testosterone [88]. The *hsd20b* gene encodes the enzyme involved in the production of 17α, 20β-dihydroxy-4-pregnen-3-one (DHP), the main progesterone for fish [89].

The genes involved in steroid hormone production in the testes of mosquitofish treated with PrP (6 and 240 μg/L) for 32d were all stimulated, including the *star*, *hsd3b*, *hsd17b3* and *hsd20b*, while the levels of *cyp11a1*, *cyp17* and *cyp19a1a* were constant. The simultaneous up-regulation of these genes would promote steroidogenesis activity and may result in the accumulation of progestogen and androgens. The results in Gal’s research supported this speculation, because PrP aggrandized the expression of *star*, as well as increased the content of testosterone and 17β-estradiol in the mouse-cultured antral follicles [90]. Sex steroid hormones are important in the regulation of fish sex differentiation, gonadal development and secondary sexual characteristics [91]. The intervention of PrP may disrupt the levels and balance of hormone homeostasis in the gonad of male mosquitofish and lead to the delayed spermatogenesis. However, future studies are warranted to verify this hypothesis, since we did not test the hormone levels.

*Shh* (sonic hedgehog) is one of the effector genes that regulate reproductive organ formation associated with hormonal signals, it can interact with function downstream of the androgenic pathway [92]. According to the literature, the Shh signaling pathway is operative and necessary in the developing prostate [93] and is indispensable for the establishment of male external genitalia characteristics [94]. Overactivation of hedgehog signaling in the developing Müllerian duct interferes with duct regression in male mice and causes subfertility [95]. Analogously, Shh protein was detected in the testis of juvenile and adult mice, and higher *shh* mRNA levels were seen in the patients with obstructive azoospermia and prostate cancer compared with the patient with cryptorchidism, suggesting that Shh signaling is involved in normal spermatogenesis [96]. In agreement with these findings, in the male mosquitofish, PrP induced transcriptional overexpression of the androgen-dependent sonic hedgehog (*shh*) and its receptor patched 1 (*ptc1*), which is likely to influence gonadal development.

In the brain, the gene transcriptions in different doses of 32d-PrP groups displayed a parabolic path, with incremental expressions in the 0.15 μg/L PrP group and a reduced level in the 240 μg/L PrP group, which may be due to the overwhelming toxic stress or related to the negative feedback loop for the action of PrP. GnRH is an important hormone in the neuroendocrine modulation of testicular development and spermatogenesis [97] via a coordinated interaction with sex steroids. Synchronized suppressions of *gnrh* and *gnrhr* mRNA expression and spermatogenesis were found in the male rats that suffered from chronic exposure to isoflurane [98]. Researchers found that GnRH steroids or agonist treatment stimulates the recovery of spermatogenesis and fertility [99]. Herein, after chronic exposure to 240 μg/L PrP for 32 d, the delayed maturity of sperm in the male mosquitofish may be associated with the inhibited expression of *gnrh* and *gnrhr*.

## 5. Conclusions

**Figure 6:**
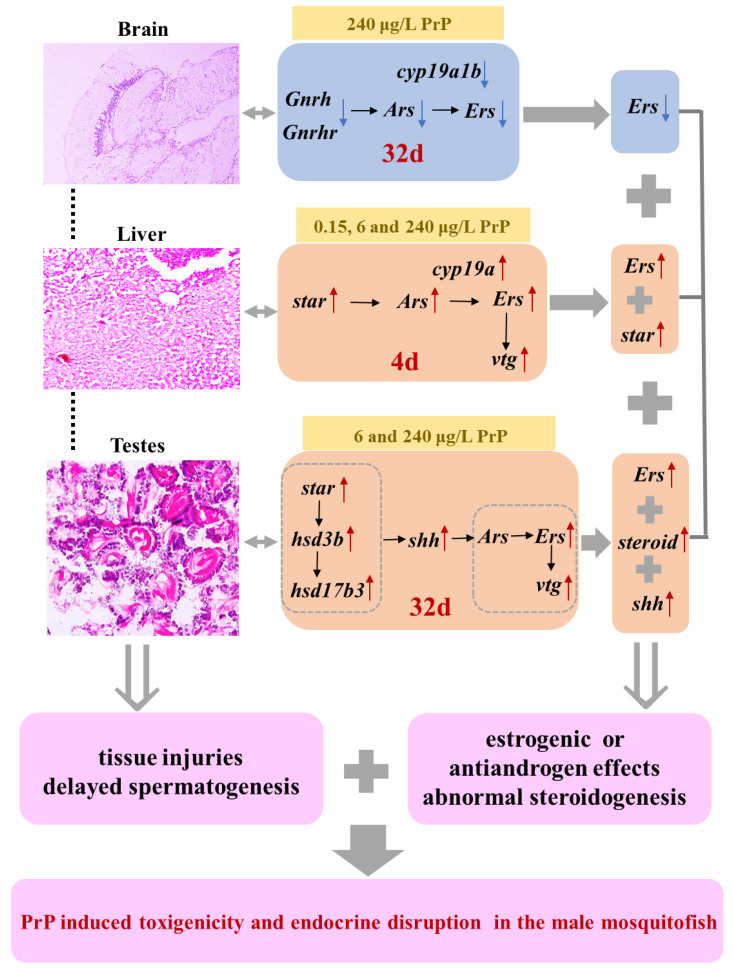
Overview of toxic effects and endocrine disruption in the male mosquitofish suffered from 0, 0.15, 6, 240 μg/L PrP for 4d and 32d. PrP exposure induced morphological injuries in the brain, liver and testes, and caused delayed spermatogenesis. The transcriptional level changes of the genes along the hypothalamus-pituitary-gonadal-liver (HPGL) axis suggested that PrP stimulated abnormal steroidogenesis, estrogenic effects or antiandrogen effects.

This study provided an integrative perspective of the endocrine interference effects of PrP on the male mosquitofish. The results systematically demonstrated that acute and chronic exposure to different concentrations of PrP caused tissue injuries in the hormone-dependent organs and delayed spermatogenesis. The tissue impairments in the brain, liver and testes presented a time- and dose-dependent manner, while the endocrine-related gene expressions varied with exposure durations, exposure concentrations and organs. The transcriptional level changes of the genes along the HPGL axis suggested that PrP stimulated abnormal steroidogenesis, estrogenic effects or antiandrogen effects (Figure 6). The findings of this work strongly indicated that PrP are a potential hazard to the physiologic functions and reproduction of adult male mosquitofish, even at an environmentally and humanly relevant dose. Considering that PrP is frequently used in daily lives and is ubiquitous in the aquatic environment, PrP safety requires special attention and further investigation. In addition, the potential risks of PrP for humans deserve scrutiny, since high concentrations of PrP were detected in different human tissues and fluids.

## Figures and Tables

**Table 1:** Histopathological effects in the brain, liver and testes of male mosquitofish suffering from 0, 0.15, 6, 240 μg/L PrP for 4d and 32d.

Tissues	Injuries	4d				32d			
		Control	0.15 μg/L	6 μg/L	240 μg/L	Control	0.15 μg/L	6 μg/L	240 μg/L
Brain	Cell cavitation	-	-	-	+	-	+	++	++
	Cytomorphosis	-	-	-	+	-	+	++	++
	Blurred cell boundaries	-	-	-	+	-	+	++	++
Liver	Hepatic sinus dilatation	-	+	++	++	-	++	++	++
	Cytoplasmic vacuolation	-	+	+	+	-	+	++	++
	Cytolysis	-	-	-	-	-	+	++	+++
	Nuclear aggregation	-	-	-	-	-	+	+	+++
Testes	Spermatorgenic cell lesion	-	-	-	-	-	+	++	+++
	Decreased mature seminal vesicle	-	-	-	-	-	++	++	+++
	Sperm cells aggregation	-	-	-	-	-	++	++	+++
	Seminiferous tubules disorder	-	-	-	-	-	++	++	+++
	Dilated intercellular space	-	-	-	-	-	++	++	+++

None or occasional (-); mild (+); moderate (++); severe (+++); n = 20.

## Data Availability

Not applicable.

## Supplementary Materials

The following supporting information can be downloaded at: https://www.mdpi.com/article/10.3390/ijerph20043557/s1, Table S1: Specific primer sequences of *Gambusia affinis* used in the qPCR experiment; Figure S1: The mRNA expression trends of endocrine-related genes in the brain of male mosquitofish suffered from different concentrations of PrP exposure for 4d and 32d; Figure S2: The mRNA expression trends of endocrine-related genes in the liver of male mosquitofish suffered from different concentrations of PrP exposure for 4d and 32d; Figure S3: The mRNA expression trends of endocrine-related genes in the testes of male mosquitofish suffered from different concentrations of PrP exposure for 4d and 32d.
