# Supplementary material for: Endocrine Disruption of Propylparaben in the Male Mosquitofish (Gambusia affinis): Tissue Injuries and Abnormal Gene Expressions of Hypothalamic-Pituitary-Gonadal-Liver Axis
Source: Int J Environ Res Public Health. 2023 Feb 17;20(4):3557. doi: 10.3390/ijerph20043557 (PMC9967665; doi:10.3390/ijerph20043557)
Supplement: Supplementary file 1 [file ijerph-20-03557-s001.zip › ijerph-2179580-supplementary.pdf]

# Endocrine disruption of propylparaben in the male mosquitofish (*Gambusia affinis*): Tissue injuries and abnormal gene expressions of hypothalamic-pituitary-gonadal-liver axis

Yun Ma[1], Yujing Li[1], Xiaohong Song[1, 2, 3, *], Tao Yang[1], Haiqin Wang[1], Yanpeng Liang [2, 3], Liangliang Huang[2, 3], Honghu Zeng[1, 2, 3, *]

[1.] College of Environmental Science and Engineering, Guilin University of Technology, Guilin, 541000, China.

[2.] Guangxi Key Laboratory of Environmental Pollution Control Theory and Technology, Guilin University of Technology, Guilin, 541000, China.

[3.] Collaborative Innovation Center for Water Pollution Control and Water Safety Guarantee in Karst Area, Guilin, 541000, China.

* Corresponding author email address: songxh@glut.edu.cn (Xiaohong Song);
zenghonghu@glut.edu.cn (Honghu Zeng)

College of Environmental Science and Engineering, Guilin University of Technology, No.12, Jiangan Road, Guilin, 541004, China.

Table S1 Specific primer sequences of *Gambusia affinis* used in the qPCR experiment.

| Gene | Forward primer (5'--3') | Reverse primer (5'--3') | Reference |
|---|---|---|---|
| *erα* | CTTGCCGACTCAGGAAGTGTTAC | TGACGCCAGTCTGTCGTTTGT | [1] |
| *erβ* | TTACTGACAGCCCATCATCCAT | GGTGGGTTTGGTTCATTGTAGAC | [1] |
| *arα* | GCTTCAGGCACGAGGATTTC | GGTGACCGCTCCGTAATGAC | [1] |
| *arβ* | CGATGCCCAGACCCAGATTAC | GAGGCGAGGTGATGAAAATGC | [1] |
| *vtgB* | TCCACCAGCATTCCATCTCAG | TAATGGCACGGACAAGGACTG | [2] |
| *vtgC* | TGAGCGACAACACTTCAGTGC | AGCCTTTGGTCCTGGGTTATC | [2] |
| *cyp19a* | ATCACATAAGATATGTCACGGTTCG | GGATGATTTGTTGCCATAGGAGC | [3] |
| *cyp19a1a* | GCCTCGTCACCAGCTCAAAG | ACCGCACCATCCATATCCAC | [2] |
| *cyp19a1b* | GAAGCTGGATGACGACCTTGACTT | GCTCCACCTTCGGGTTTTGTTT | [2] |
| *cyp11a1* | CAGGTTGAGTATGTGGCGGA | GGATGATGCTGCTGCTCTCT | [2] |
| *cyp17* | TGGTTCTGTTCTCCAGTCGG | AGAGCAGGTGAGGAGGAAGG | [2] |
| *gnrh* | TGTCGAAACGCTGACTCTGT | CAGTTCCCTCTTTCCGCCTG | [2] |
| *gnrhr* | TGACGTTTGTGGTGATGCCT | CAAGCTGATGACGACCAGGA | [2] |
| *hsd3b* | TTCCACATCGCTTCCCTCAT | TCCTCGGTGCCGTTGATTAT | [2] |
| *hsd17b3* | CTGGGTGCAGTAGTTGTCGT | GCCCATTCTCCCATAGAAGTGA | [2] |
| *hsd20b* | AAGGTGGAGACCAGGACGAT | CCAGATTAGCCAGTCCTTCCTC | [2] |
| *shh* | TGTGGCGGAGAAGACCCTG | GAGCTTCACCCCAGGCCAT | [2] |
| *ptc1* | GACGGCTGGCATCATTGTGTT | GGAGATTGCACCGTCAAGCACT | [2] |
| *star* | TAGTGGGACCGAGGGACTTT | ACACCTTTCTGCTCTGGCAT | [2] |
| *gapdh* | TTCACGCCATCACTGCCACA | TCAGGGATGACCTTGCCAACAG | [4] |

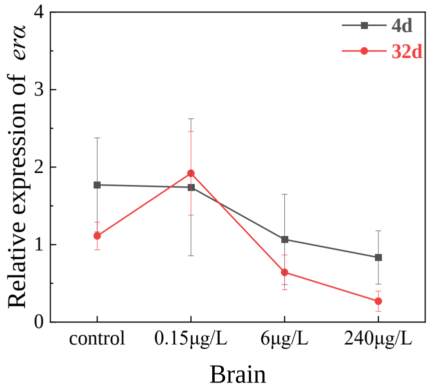


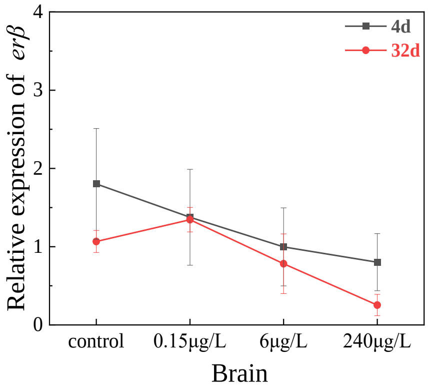

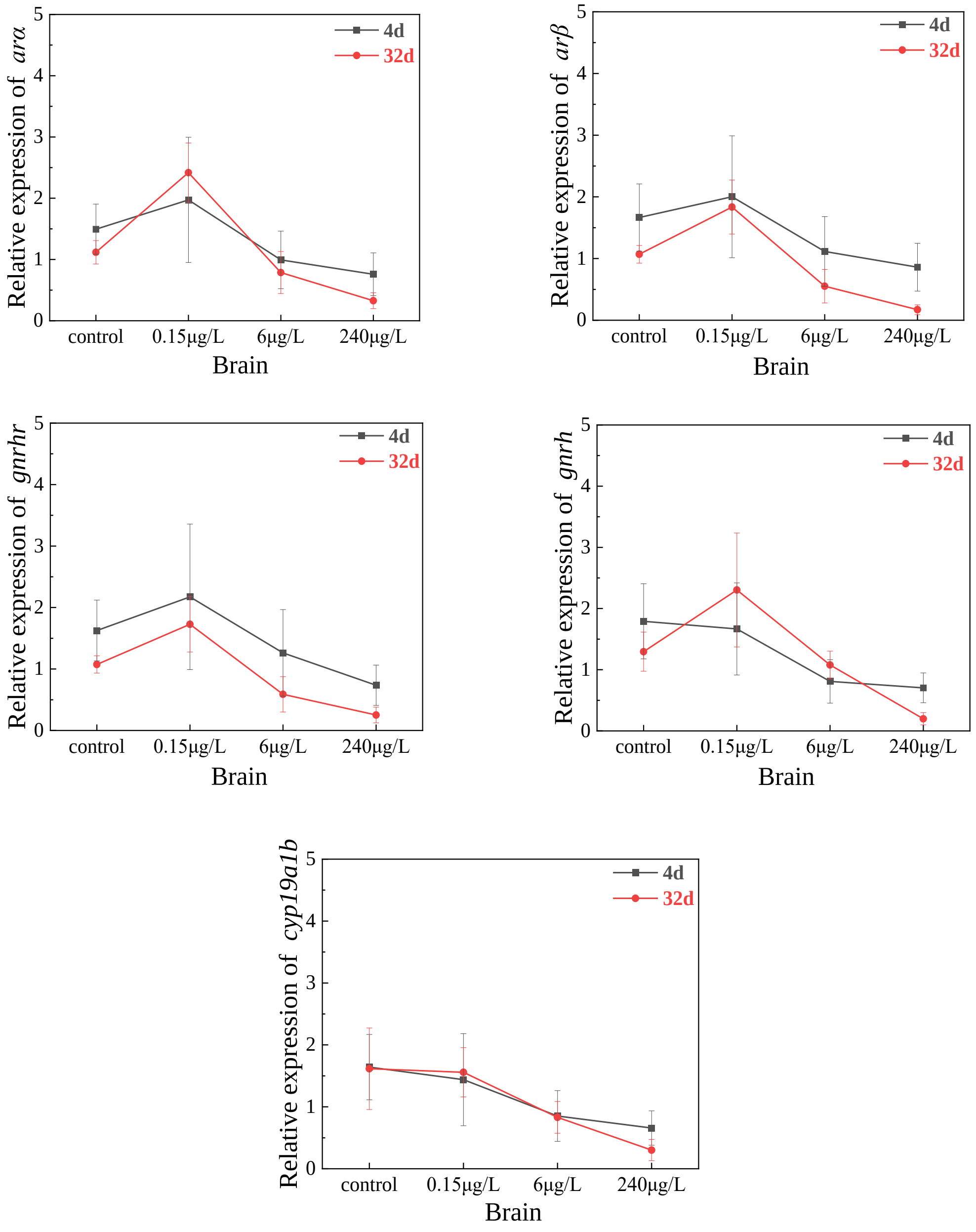


**Figure S1** The mRNA expression trends of endocrine-related genes in the brain of male mosquitofish suffered from different concentrations of PrP exposure for 4d and 32d.

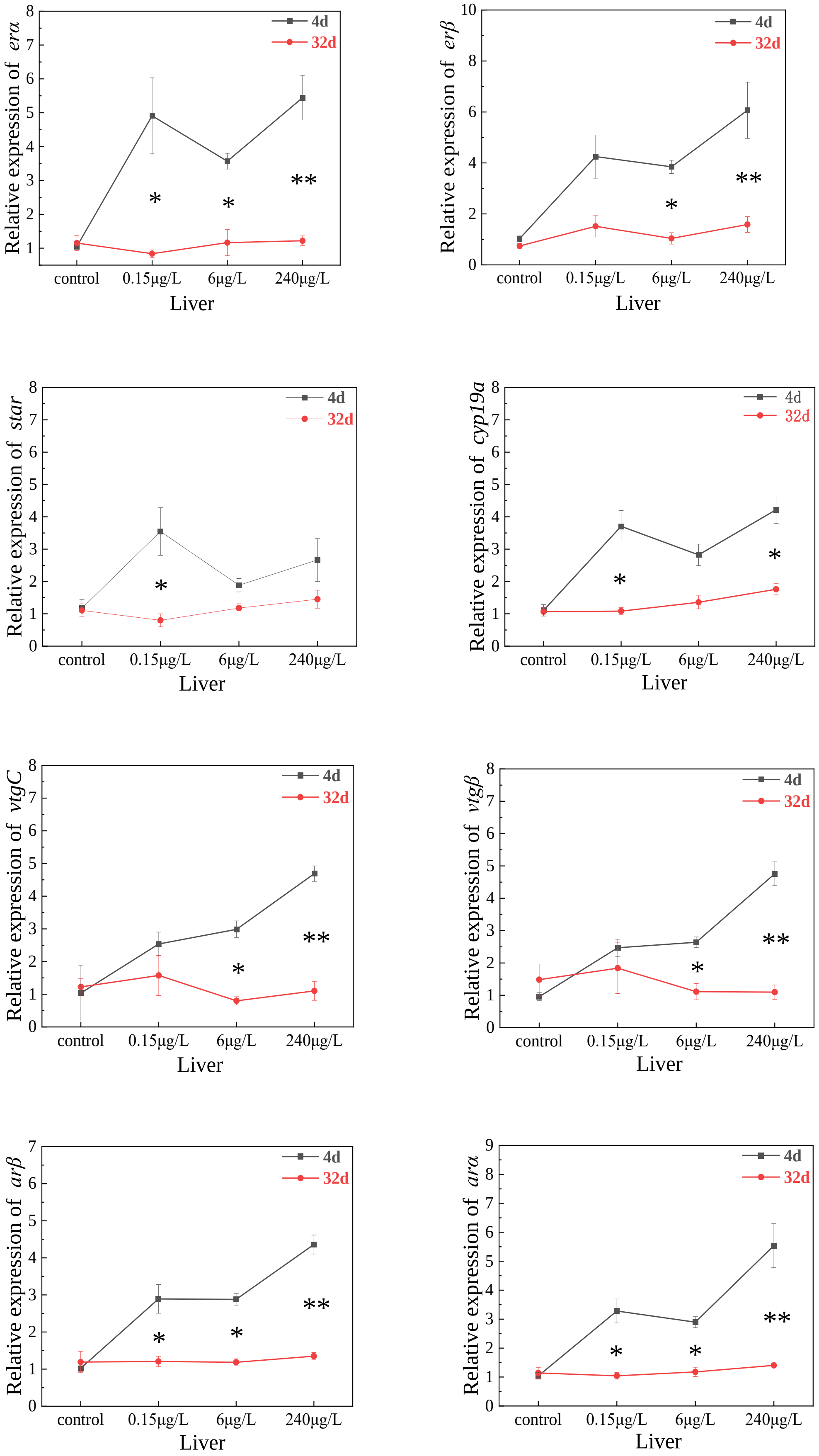

Relative expression of era
4d
32d
*
*
**
control
0.15μg/L
6μg/L
240μg/L
Liver
Relative expression of erβ
4d
32d
*
**
control
0.15μg/L
6μg/L
240μg/L
Liver
Relative expression of star
4d
32d
*
control
0.15μg/L
6μg/L
240μg/L
Liver
Relative expression of cyp19a
4d
32d
*
*
control
0.15μg/L
6μg/L
240μg/L
Liver
Relative expression of vtgC
4d
32d
*
**
control
0.15μg/L
6μg/L
240μg/L
Liver
Relative expression of vtgβ
4d
32d
*
**
control
0.15μg/L
6μg/L
240μg/L
Liver
Relative expression of arβ
4d
32d
*
*
**
control
0.15μg/L
6μg/L
240μg/L
Liver
Relative expression of ara
4d
32d
*
*
**
control
0.15μg/L
6μg/L
240μg/L
Liver

**Figure S2** The mRNA expression trends of endocrine-related genes in the liver of male mosquitofish suffered from different concentrations of PrP exposure for 4d and 32d. Data were analyzed by *t*-test and Tukey's multiple comparisons. Asterisks (*) indicate statistically significant differences between the genes at two time points with the same concentrations (* $p$ <0.05, ** $p$ <0.01).

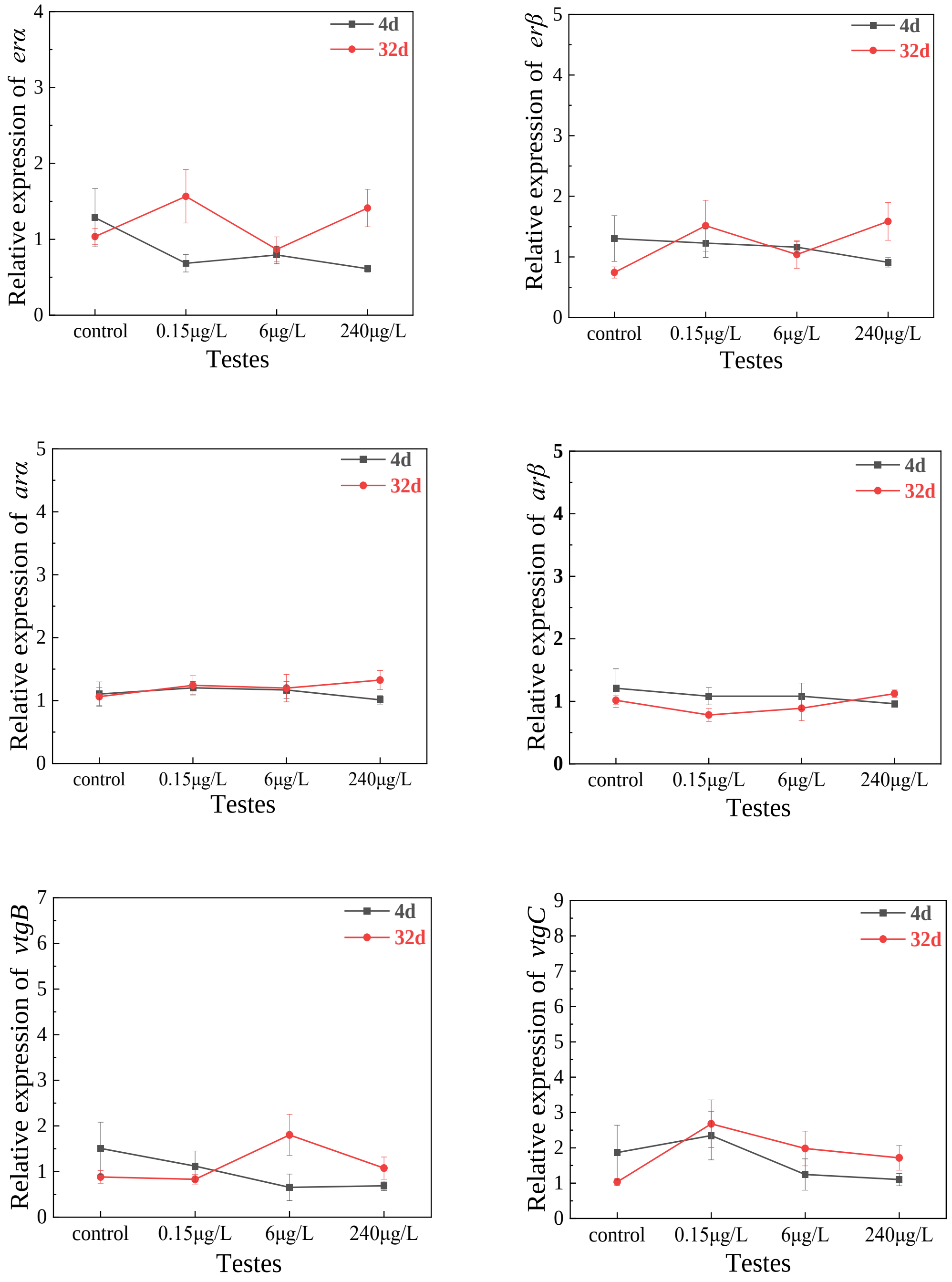

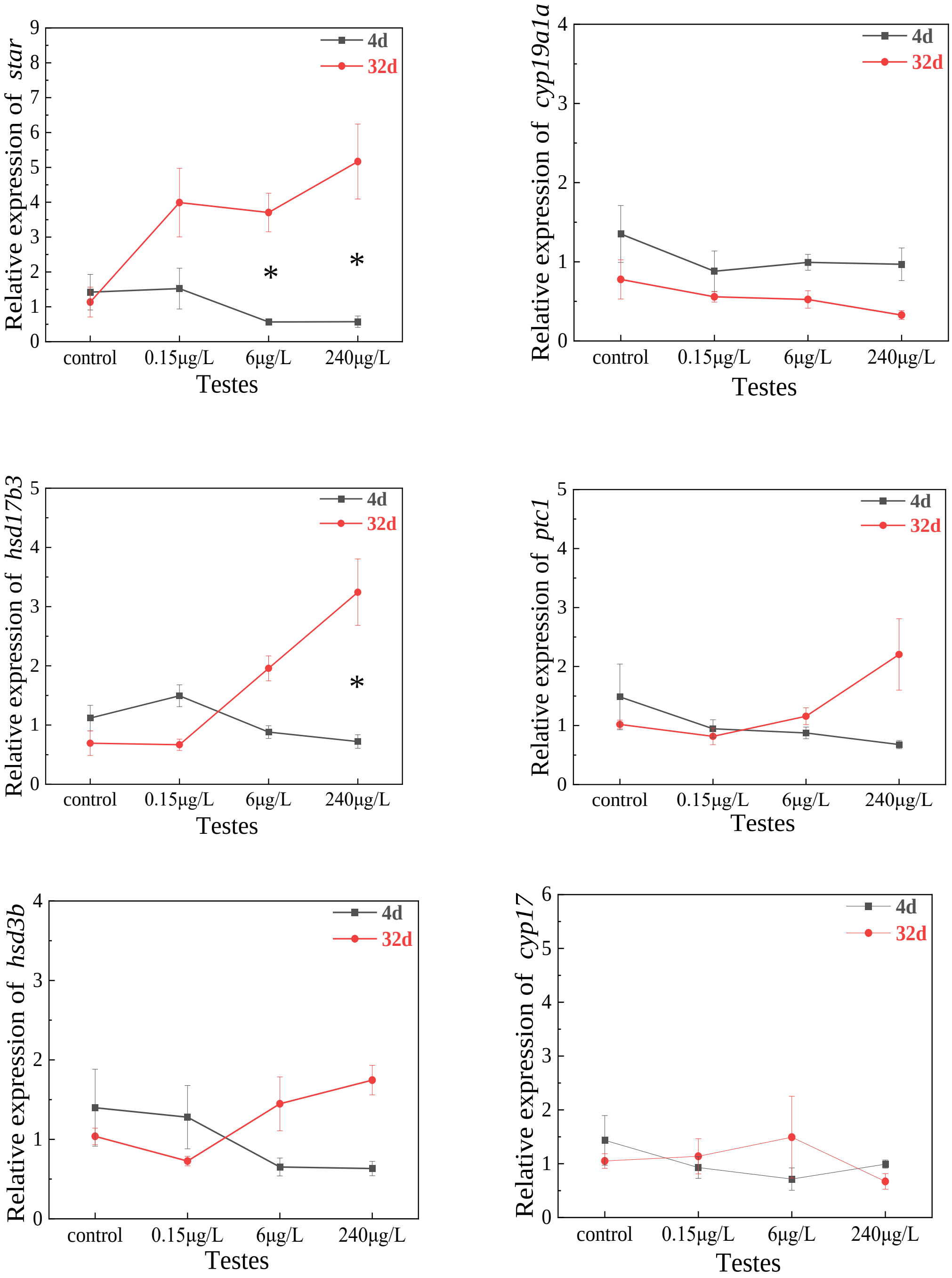

Relative expression of star
Relative expression of cyp19a1a
Relative expression of hsd17b3
Relative expression of ptc1
Relative expression of hsd3b
Relative expression of cyp17
4d
32d
*
control
0.15μg/L
6μg/L
240μg/L
Testes

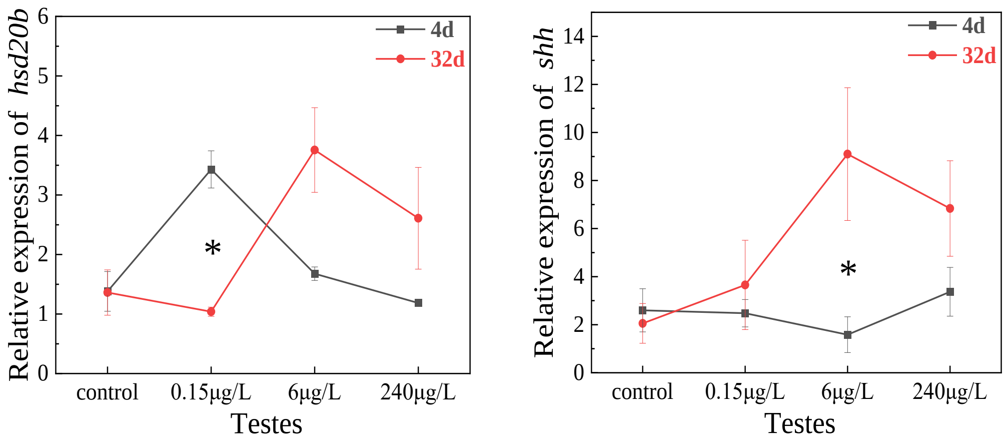


**Figure S3** The mRNA expression trends of endocrine-related genes in the testes of male mosquitofish suffered from different concentrations of PrP exposure for 4d and 32d. Data were analyzed by *t*-test and Tukey's multiple comparisons. Asterisks (*) indicate statistically significant differences between the genes at two time points with the same concentrations (* $p$ <0.05, ** $p$ <0.01).

## References


1. Zhong, L.; Liang, Y. Q.; Lu, M.; Pan, C. G.; Dong, Z.; Zhao, H.; Li, C.; Lin, Z.; Yao, L. Effects of dexamethasone on the morphology, gene expression and hepatic histology in adult female mosquitofish (*Gambusia affinis*). *Chemosphere* **2021**, 274, 129797. https://doi.org/10.1016/j.chemosphere.2021.129797.
2. Fang, G.-Z.; Huang, G.-Y.; Ying, G.-G.; Qiu, S.-Q.; Shi, W.-J.; Xie, L.; Yang, Y.-Y.; Ma, D.-D. Endocrine disrupting effects of binary mixtures of 17β-estradiol and testosterone in adult female western mosquitofish (*Gambusia affinis*). *Ecotoxicol. Environ. Saf.* 2021, 208, 111566. https://doi.org/10.1016/j.ecoenv.2020.111566.
3. Hou, L.; Chen, S.; Chen, H.; Ying, G.; Chen, D.; Liu, J.; Liang, Y.; Wu, R.; Fang, X.; Zhang, C.; Xie, L. Rapid masculinization and effects on the liver of female western mosquitofish (*Gambusia affinis*) by norethindrone. *Chemosphere* 2019, 216, 94-102. https://doi.org/10.1016/j.chemosphere.2018.10.130.
4. Ou, R.; Wu, X.; Peijia, K.; Lan, W.; Tian, S.; Liang, X.; Nie, X. Cloning of cat, gapdh and gst genes of *Gambusia affinis* and its application in ecotoxicology. *Asian Journal of Ecotoxicology* 2015, 10, (3), 83-92. DOI: 10． 7524 / AJE． 1673-5897-20140413003.
